# Entomological survey in two communes with residual malaria transmission in Gia Lai Province in the central highlands of Vietnam

**DOI:** 10.1186/s12936-021-03941-6

**Published:** 2021-10-16

**Authors:** Thai Quang Nguyen, Manh Duc Nguyen, Vinh Xuan Pham, Huan Mah Ro, Michael D. Edstein, Weng K. Chow, Nicholas J. Martin, Jeffrey C. Hertz, Maysa T. Motoki

**Affiliations:** 1 Vietnam People’s Army Military, Institute of Preventive Medicine, Hanoi, Vietnam; 2 Gia Lai Center for Disease Control (CDC), Pleiku, Gia Lai Vietnam; 3 Australian Defence Force Malaria and Infectious Disease Institute, Brisbane, Australia; 4 US Naval Medical Research Unit-Two (NAMRU-2), Singapore, Singapore; 5 Vysnova Partners Inc, Landover, MD USA; 6 Department of Entomology, Natural Museum of Natural History, Smithsonian Institution, Museum Support Center, Suitland, MD USA

**Keywords:** *Anopheles* diversity, Host-seeking activity, Malaria, Vietnam

## Abstract

**Background:**

In 2018, the National Malaria Control Programme in Vietnam switched from prioritizing malaria control to elimination. However, with the ongoing elimination programme, there are still areas where residual malaria transmission persists, including the central highlands. This entomological survey was conducted to evaluate *Anopheles* diversity and host-seeking activity of *Anopheles* vectors in two communes with very low malaria transmission in Gia Lai Province.

**Methods:**

*Anopheles* species were collected in Ia DReh commune and Ia KDam commune, Gia Lai Province in the central highlands of Vietnam. Collections were conducted using human-baited double net trap, light trap and manual aspiration collections around cattle sheds, in the dry and rainy season. Mosquito specimens were identified morphologically, and members of species complexes were distinguished molecularly. Mosquito night-feeding patterns were investigated during the dry and rainy seasons.

**Results:**

Overall, 18,835 specimens including 19 taxa were collected in Ia KDam and Ia DReh communes. These included the primary malaria vectors, *Anopheles dirus* and *Anopheles minimus*, and other secondary vector species. *Anopheles dirus* was observed to be an anthropophilic species, whereas *An. minimus* and a number of secondary vectors were observed to be zoophilic. *Anopheles vagus* was the dominant species, followed by *Anopheles sinensis* and *Anopheles peditaeniatus.* The majority of specimens were collected in the rainy season due to the relatively large number of *An. vagus*, while *An. peditaeniatus*, *An. dirus, Anopheles kochi*, *Anopheles monstrosus* and *Anopheles tessellatus* were collected in greater numbers during the dry season. The peak of host-seeking activity for *An. dirus*, *An. sinensis*, and *An. vagus* was between 18.00 and 19.00 h.

**Conclusion:**

This study provided information on the diversity, seasonal prevalence and behaviour of *Anopheles* at the study sites. Identifying the diverse mosquito fauna in the central highlands of Vietnam allows species-specific control measures to be implemented by the National Programme to reduce malaria in areas of very low malaria transmission. The peak *Anopheles* host-seeking activity observed in this study was between 18.00 and 23.00 h, which highlights the need to better characterize *Anopheles* behaviour in this region of Vietnam to inform on vector control strategies.

**Supplementary Information:**

The online version contains supplementary material available at 10.1186/s12936-021-03941-6.

## Background

Currently, the primary goal of Vietnam’s National Malaria Control Programme (NMCP) is to eliminate malaria by 2030 [1]. The NMCP recommends the use of insecticide-treated nets (ITNs), long-lasting insecticide nets (LLINs), social mobilization, targeted residual indoor spraying, and improved anti-malarial drug treatment. These strategies significantly reduced the spread of malaria in many areas in the country [2–4]. However, despite these measures, there remain residual malaria transmission areas, predominately in forested regions of central and southern Vietnam [5–10]. These very low malaria transmission areas harbour primary malaria vectors and there are reports that long-lasting insecticidal net (LLIN) use is less common [11, 12]. In deforested rural areas, where land has been cleared for cultivation, many secondary malaria vectors are present and may contribute to maintaining malaria transmission outside of forested areas [10].

Some members of the *Anopheles dirus* complex, Maculatus Group, and Hyrcanus Group have been recognized as malaria vectors in Southeast Asia [10, 13]. There are over 64 *Anopheles* species in Vietnam [14]. *Anopheles dirus*, *Anopheles minimus, Anopheles epiroticus,* and *Anopheles sundaicus* have been identified as the primary malaria vectors [10, 13, 15, 16], while many other species (*Anopheles aconitus*, *Anopheles barbirostris*, *Anopheles campestris*, *Anopheles harrisoni*, *Anopheles indefinitus*, *Anopheles jeyporiensis*, *Anopheles maculatus*, *Anopheles nimpe*, *Anopheles nivipes*, *Anopheles peditaeniatus*, *Anopheles philippinensis*, *Anopheles sawadwongporni*, *Anopheles sinensis*, *Anopheles subpictus*, and *Anopheles vagus*) are considered secondary malaria vectors [10, 16].

Correctly identifying the mosquito fauna in malaria-endemic areas is critical to implementing species-specific control measures and other public health interventions. The identification of mosquito vectors is mainly based on morphological diagnostic characters. However, misidentifications of closely related species, with overlapping morphological characters, is possible and is more likely amongst sibling vector species [17]. In these circumstances, molecular identification has been used for definitive identification [18–21].

In Gia Lai Province, in the central highlands of Vietnam, there are many individuals who depend on the forest, forest fringe and deforested areas for subsistence (agriculture and wood cutting activities) with potential exposure to malaria vectors [22]. In 2020, Krong Pa was the most impacted district with 459 malaria cases, while 43 cases were reported in Ia Pa District (Gia Lai Province Center for Disease Control and Prevention—CDC pers. comm.). A survey of *Anopheles* vectors was conducted in 2019 to inform vector control strategies in residual malaria transmission areas of Gia Lia Province. The study focused on characterizing diversity and host-seeking activity of *Anopheles* vectors in Gia Lai Province.

## Methods

### Study sites

**Fig. 1:**
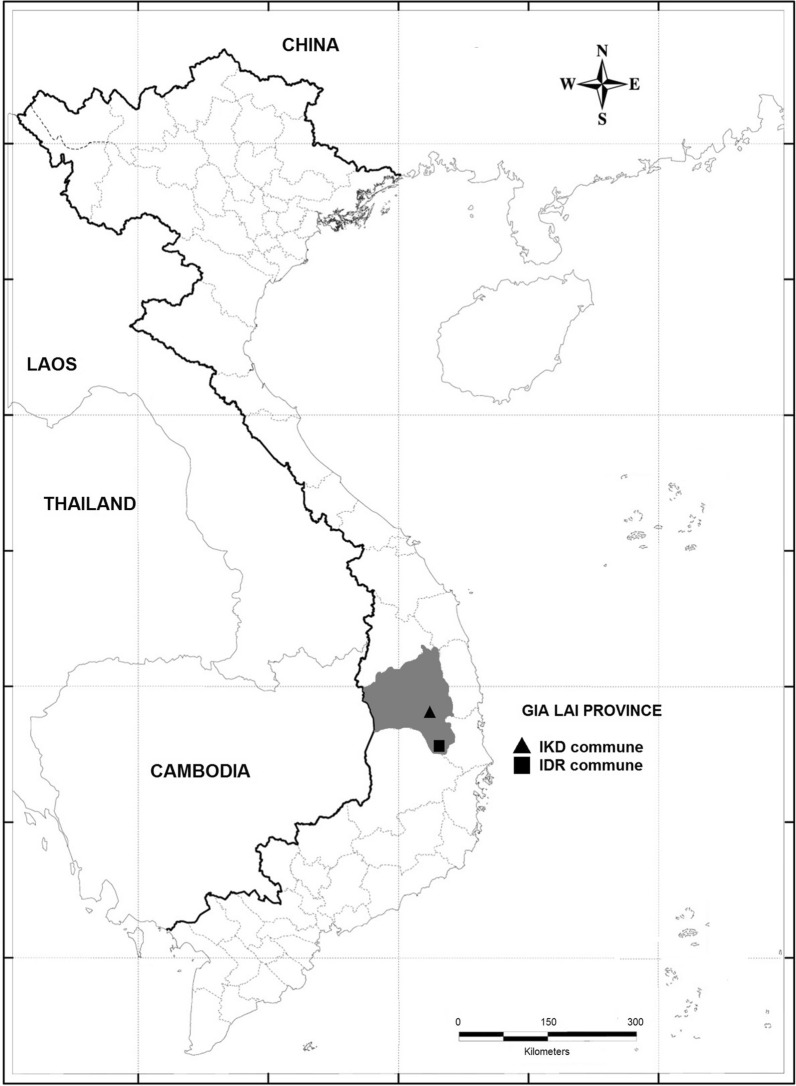
Map of study sites in Gia Lai Province, Vietnam. *IKD* Ia KDam commune; Ia Pa district; *IDR* Ia DReh commune, Krong Pa district

Gia Lai is a mountainous province in the northern region of the central highlands of Vietnam (Fig. 1), and has a tropical monsoon climate, and two distinctive seasons: the rainy season (May to October) and the dry season (November to April). The average annual rainfall is 2100 mm to 2200 mm, the average temperature is 22 °C to 25 °C and the average annual air humidity is about 80% (Gia Lai Province Local Health Office, pers. comm.).

Entomological surveys were conducted during the rainy and dry seasons in Ia DReh (IDR) commune, Krong Pa District, and in Ia KDam (IKD) commune, Ia Pa District, Gia Lai Province (Fig. 1). Village sites (inside the villages) are those where people live. Village sites contain houses and small gardens with few trees; whereas farm (mixed with cassava, sugar cane and watermelon plantations) and forest sites (tropical dense forest in IDR and deforested area in IKD) are places where villagers conduct agriculture and forestry activities, but are not permanent residences (TQN pers. obsv.). IDR commune is 110–140 m above sea level and includes 8 villages: Hvut, Nai, Drai, Hyu, Djrong, Tring, Bau, and Chum Kia. There is a nature reserve named Ea So, which is comprised of dense tropical forest approximately 10 km from the villages. IKD commune is located 232 m above sea level, and includes deforested and open areas used for farming purposes. This commune comprises 7 villages: Hbel, Plei Toan 1, Plei Toan 2, Plei KDam 1, Plei Kdam 2, Bau, and Chroh Ko. In 2020, the population was 5,363 people in IDR (2642 women, 2721 men) and 4176 people in IKD (2341 women, 1835 men). In both communes, 30% of population were between 15 and 49 years old. In the last years, the strategies of vector control have been the use of LLINs and insecticide-treated nets (ITNs), where IDR has been supplied with LLINs, and IKD with LLINs and ITNs (Gia Lai Province, CDC pers. comm.).

In 2019, 103 and 131 symptomatic malaria cases confirmed by blood film microscopy were reported in IDR and IKD communes, respectively (Gia Lai Province, CDC pers. comm.). The annual parasite incidence was 21.2 (103/4.857) cases per 1000 populations for the IDR commune and 35.2 (131/3.717) cases per 1000 populations for the IKD commune. Based on the annual parasite incidence < 100 cases per 1000 populations, both communes would be classified as areas of very low malaria transmission [23].

### *Anopheles* collection

Entomology surveys were conducted in the dry season (1–28 January, 2019) and in the rainy season (29 June-24 July, 2019). During each survey, mosquitoes were collected in both communes, IDR and IKD, using light traps (LT) from 18.00 to 06.00 h, human-baited double net traps (HDN) [24], and using manual aspirations around cattle sheds (CS) from 18.00 to 24.00 h. HDN and CS collections were conducted by one collector (i.e., one collector per trap/method).

For surveys in villages sites, during each season, one indoor HDN and one outdoor HDN was set up for 18 nights; CS collections were conducted for 24 nights; and LT collections were set up indoors (n = 2) and outdoors (n = 2) for 18 nights in IDR and IKD. For surveys in farm and forest sites, one outdoor HDN was set up for 10 nights at IDR and IKD during each season; CS collections were conducted for two nights during the rainy season in IDR, whereas in IKD were conducted for three nights during the dry season and for four nights during the rainy season; no LT was set up in IDR, whereas two LTs were set up in IKD for five nights only during the rainy season.

### *Anopheles* identification

Mosquitoes were morphologically identified under stereomicroscopes using keys developed by the National Institute of Malariology, Parasitology and Entomology [25]. Molecular methods were used for the identification of species complex/group members, except Barbirostris Complex was not identified molecularly, it was identified by morphological characters as *An. barbirostris *sensu lato (s.l.). DNA from head and prothorax of collected mosquito specimens was extracted using the QIAamp DNA Mini Kit—DNA Purification from Tissues as described in the manufacturer’s instructions (Qiagen, Hilden, Germany). To identify species complex or group of *Anopheles*, the molecular methods used are those described by Garros et al. for the Funestus Group [26]; Huong et al. for the Dirus Complex [27]; and Walton et al. for the Maculatus Group [20]. Specimen identification was recognized according to the length of the product for each polymerase chain reaction (PCR)-based identification (Table 1).

**Table 1:** Primers used in this study for the molecular identification of *Anopheles* complex/group members in village and farm/forest sites in Ia DReh commune, Krong Pa district, and Ia KDam commune, Ia Pa district, Gia Lai Province, Vietnam

Species	Primer name	Sequence (5ʹ-3ʹ)	PCR size	References
Universal forward primer	ITS2A	5ʹ TGT GAA CTG CAG GAC ACA T 3ʹ		Garros et al. [26]
*An. minimus*	MIA	5ʹ CCC GTG CGA CTT GAC GA 3ʹ	310-bp	Garros et al. [26]
*An. harrisoni*	MIC	5ʹ GTT CAT TCA GCA ACA TCA GT 3ʹ	180-bp	Garros et al. [26]
*An. aconitus*	ACO	5ʹ ACA GCG TGT ACG TCC AGT 3ʹ	200-bp	Garros et al. [26]
*An. varuna*	VAR	5ʹ TTG ACC ACT TTC GAC GCA 3ʹ	260-bp	Garros et al. [26]
*An. pampanai*	PAM	5ʹ TGT ACA TCG GCC GGG GTA 3ʹ	90-bp	Garros et al. [26]
*An. dirus* (A)	FA2	5ʹ TCG GGT TCT ATA ATA TTC GCT 3ʹ	120-bp	Huong et al. [27]
	RA2	5ʹ GAC CTA GTG TTT GGG AAG GT3ʹ		
*An. cracens*	FB2	5ʹ GCT TCA AGA CCA AAA CCA TCA3ʹ	75-bp	Huong et al. [27]
	RB2	5ʹ GAA TTT ACA ACT TTT GAC CTG G3ʹ		
*An. scanloni* (C)	FC3	5ʹ ATT CTG TGC CAA AAT TGT ACC T3ʹ	60-bp	Huong et al. [27]
	RC3	5ʹ TTG TCC GAA ACT GGC TTC T 3ʹ		
*An. baimaii*	FD1	5ʹ AGG GCA CAA AAG TTA TTA ACT T 3ʹ	172-bp	Huong et al. [27]
	RD1	5ʹ GTG AAG AGC GAA TAT TGT AGC 3ʹ		
Universal forward primer	5.8F	5ʹ ATC ACT CGG CTC GTG GAT CG 3ʹ		Walton et al. [20]
*An. maculatus*	MAC	5ʹ GAC GGT CAG TCT GGT AAA GT 3ʹ	180-bp	Walton et al. [20]
*An. pseudowillmori*	PSEU	5ʹ GCC CCC GGG TGT CAA ACA G 3ʹ	203-bp	Walton et al. [20]
*An. sawadwongporni*	SAW	5ʹ ACG GTC CCG CAT CAG GTG C 3ʹ	242-bp	Walton et al. [20]
*An. rampae* (form k)	K	5ʹ TTC ATC GCT CGC CCT TAC AA 3ʹ	301-bp	Walton et al. [20]
*An. dravidicus*	DRAV	5ʹ GCC TAC TTT GAG CGA GAC CA 3ʹ	477-bp	Walton et al. [20]

For the PCR-based identification for the Funestus Group [26], each PCR contained 1.8 µl of DNA template, 12.5 µl of PCR master mix 2x (containing 0.5 units of Taq DNA polymerase, 200 µM of dNTPs, 2.5 mM of MgCl_2_), 0.16 µM of each primer (ITS2A, MIA, MIC, ACO, VAR, PAM) (Table 1) made up to a total volume of 25 µl using double distilled water (ddH_2_O). The PCR cycle was 94 °C for 2 min, 40 cycles of 94 °C for 30 s, 45 °C for 30 s and 72 °C for 40 s, 1 cycle of 72 °C for 5 min and a 10 °C hold. Five µl of the product was then run on 3% gel agarose and the results visualized by ultraviolet (UV) light.

For the PCR-based identification for the Dirus Complex [27], each PCR performed (a total of 50 µl), contained 200 μM dNTPs each, 5 μl of 10 × PCR buffer (100 mM Tris–HCl, 500 mM KCl, 15 mM MgCl2, 0.1% gelatin; pH 9.5), 1 µM (FA2/RA2, FD1/RD1), 1 µM (FB2/RB2), 1 µM (FC3/RD3) of each primer, and 5 µl of DNA template. The mixture was preheated for 2 min at 94 °C, then 1 µl of *Taq* DNA polymerase was added at 72 °C followed by 30 cycles of amplification at 95 °C for 15 s, 60 °C for 15 s, 72 °C for 15 s and a final extension step at 72 °C for 5 min. Five µl of the product was then run on 3% gel agarose with the results visualized by UV light.

For PCR-based identification for the Maculatus Group [20], each PCR contained 1 µl of DNA template, 12.5 µl of PCR master mix 2x (containing 0.5 units of Taq DNA polymerase, 200 µM of dNTPs, 2.5 mM of MgCl_2_), 0.4 µM of each primer (5.8F, MAC, DRAV, K, SAW, PSEU) [20] made up to a total volume of 25 µl using ddH_2_O. The PCR cycle was 95 °C for 15 min, 35 cycles of 94 °C for 1 min, 61 °C for 30 s and 72 °C for 30 s, 1 cycle of 72 °C for 5 min and a 10 °C hold. Five µl of the PCR product was run on 3% gel agarose and the results visualized by UV light.

### Statistical analysis

Considering the heterogeneous number of traps and number of collections, specific comparisons of each collection method between communes, and according to the season were employed. For statistical analysis, the Monte Carlo Method, or Fisher’s Exact Test were used when the sample size was small. These statistical tests were conducted using IBM SPSS statistics, version 25, with statistical significance defined as a p < 0.05.

### Study protocol review

The Institute of Malariology, Parasitology and Entomology Quy Nhon Institutional Review Board (IMPE-QN IRB) determined this study to be exempt from the IRB review (number 16/VSR-NCDT); the determination was made based on the national guideline of the Vietnamese Ministry of Health IRB on ethics in biomedical research. The study protocol # HRPO.NAMRU2.2020.0002—HDN Trap was reviewed by Naval Medical Research Center- Asia Human Research Protections Review Board and is in compliance with all applicable Federal regulations governing the protection of human subjects.

## Results

### *Anopheles* diversity

Overall, 18,835 mosquitoes were collected, including 14 taxa in IDR (n = 9553) and 18 taxa in IKD (n = 9282) (Table 2). Members of the Funestus Group were molecularly identified as *An. minimus *sensu stricto (s.s.), *An. aconitus*, *An. harrisoni*, *An. varuna*, and *An. pampanai*. Three samples could not be identified to species. For the Maculatus Group, *An. maculatus* s.s., *An. sawadwongporni* and *An. rampae* were identified. For the Dirus Complex, only *An. dirus* s.s. was recognized, and one sample could not be identified to species (Table 2).

**Table 2:** *Anopheles* specimens identified morphologically and molecularly in village and farm and forest sites in Ia DReh commune, Krong Pa district, and in Ia KDam commune, Ia Pa district, Gia Lai Province, Vietnam

Group/complex	Species	Communes			
		IDR		IKD	
		Morp. ID	Mol. ID	Morp. ID	Mol. ID
Annularis group	*An. philippinensis*^b^	7	–	4	–
Barbirostris complex	*An. barbirostris* s.l.^b^	20	–	121	–
Dirus complex	*An. dirus*^a^	86	85	5	5
	N/I	0	1	0	–
Funestus group	*An. aconitus*^b^	53	53	97	92
	*An. minimus*^a^	0	–	143	119
	*An. harrisoni*^b^	0	–	0	3
	*An. varuna*	0	–	0	21
	*An. pampanai*	0	–	0	2
	N/I	0	–	0	3
Hyrcanus group	*An. peditaeniatus*^b^	373	–	378	–
	*An. sinensis*^b^	534	–	412	–
Jamesii group	*An. jamesii*	9	–	27	–
	*An. splendidus*	14	–	1	–
Kochi group	*An. kochi*	73	–	–	–
Maculatus group	*An. maculatus*^b^	23	1	224	5
	*An. sawadwongporni*^b^	0	22	0	218
	*An. rampae*	0	–	0	1
Subpictus group	*An. vagus*^b^	8016	–	7643	–
Tessellatus group	*An. tessellatus*	163	–	87	–
No Group/complex	*An. monstrosus*	182	–	140	–
	Total	9553		9282	

*IDR* Ia DReh commune, *IKD* Ia KDam commune, *Morp. ID* morphological identification, *Mol. ID* molecular identification, *N/I* could not be identified molecularly

^a^Main malaria vectors

^b^Secondary malaria vectors according to the WHO [16], and Do Manh et al. [10]

*Anopheles kochi* was found only in IDR, while *An. harrisoni*, *An. minimus*, *An. pampanai*, *An. rampae* and *An. varuna* were unique to IKD (Table 2). *Anopheles dirus* were more common in IDR, while *An. barbirostris* s.l. and *An. sawadwongporni* were more common in IKD (Table 2). The three most common species collected in both communes were *An. vagus*, *An. sinensis* and *An. peditaeniatus* (Table 2).

### Density and comparison of mosquitoes according to each collection method

*Anopheles vagus* was collected in high density using CS (85.0%, 15,193/17,871), HDN (48.8%, 230/471), and LT (47.9%, 236/493) (Tables 3–5). The remaining *Anopheles* collected by CS included 18 taxa, ranging from 1 to 831 specimens (Table 3). In the village site in IDR, CS collected more mosquito specimens (54.4%, 8909/16,376) than in IKD (45.6%, 7467/16,376) (p < 0.05, Monte Carlo Method) (Table 3). Due to the heterogeneity of the number of collection traps and bad weather conditions, no comparison was made in farm/forest sites.

**Table 3:** Number and percentage of *Anopheles* collected using CS in village and farm and forest sites in IDR and IKD communes

Species	Ia DReh		Ia KDam		Total	%
	V	F	V	F		
*An. vagus*	7649	251	6687	606	15,193	85.0
*An. sinensis*	489	20	308	14	831	4.65
*An. peditaeniatus*	324	3	279	10	616	3.45
*An. monstrosus*	174	–	131	1	306	1.71
*An. sawadwongporni*	3	–	–	199	202	1.13
*An. tessellatus*	128	1	61	11	201	1.12
*An. barbirostris* s.l	17	–	–	119	136	0.76
*An. aconitus*	37	–	–	85	122	0.68
*An. minimus*	–	–	–	111	111	0.62
*An. kochi*	67	–	–	–	67	0.37
*An. jamesii*	9	–	–	25	34	0.19
*An. varuna*	–	–	–	20	20	0.11
*An. splendidus*	8	1	–	1	10	0.06
*An. philippinensis*	4	–	1	3	8	0.04
*An. maculatus*	–	–	–	5	5	0.03
*An. harrisoni*	–	–	–	3	3	0.02
*Anopheles* sp.	–	–	–	3	3	0.02
*An. pampanai*	–	–	–	1	1	0.01
*An. rampae*	–	–	–	1	1	0.01
*An. dirus*	–	–	–	1	1	0.01
Total	8909	276	7467	1219	17,871	100

*CS* collection around cattle shed, *V* village sites, *F* farm and forest sites

In addition to *An. vagus* collected using HDN, *An. dirus* represented 18.7% (88/471), *An. sinensis,* 8% (38/471)*, An. tessellatus,* 7.0% (33/471), *An. peditaeniatus* and *An. sawadwongporni,* 6.6% (31/471) of each species, with the remaining mosquitoes collected belonging to the other species listed in Table 4. In village sites, HDN collected more mosquito specimens in IKD (74.6%, 156/209) than in IDR (25.4%, 53/209) (p < 0.05, Fisher Exact Test), while in farm and forest sites, mosquito specimens were collected more in IDR (56.9%, 149/262) than in IKD (43.1%, 113/262) (p < 0.05, Fisher Exact Test) (Table 4).

**Table 4:** Number and percentage of *Anopheles* collected using HDN in village and farm and forest sites in IDR and IKD communes

Species	Ia DReh		Ia KDam		Total	%
	V	F	V	F		
*An. vagus*	40	7	115	68	230	48.8
*An. dirus*	–	85	–	3	88	18.7
*An. sinensis*	4	–	26	8	38	8.0
*An. tessellatus*	–	22	2	9	33	7.0
*An. sawadwongporni*	–	19	–	12	31	6.6
*An. peditaeniatus*	7	1	12	10	30	6.5
*An. aconitus*	–	9	–	1	10	2.0
*An. barbirostris* s.l	–	2	–	–	2	0.5
*An. monstrosus*	–	1	1	–	2	0.5
*An. kochi*	1	–	–	–	1	0.2
*An. maculatus*	–	1	–	–	1	0.2
*An. minimus*	–	–	–	1	1	0.2
*An. pampanai*	–	–	–	1	1	0.2
*An. philippinensis*	–	1	–	–	1	0.2
*An. splendidus*	1	–	–	–	1	0.2
*Anopheles* sp.	–	1	–	–	1	0.2
Total	53	149	156	113	471	100

*HDN* human-baited double net trap, *V* village sites, *F* farm and forest sites

The LT collected 493 specimens. In addition to *An. vagus*, 21.3% (105/493) were *An. peditaeniatus*, and 15.6% (77/493) were *An. sinensis*. The remaining 13.2% of specimens collected in LT included 12 additional species at low collection densities (Table 5). *Anopheles kochi* were found only in the village sites (Tables 3–5); while *An. dirus*, *An. harrisoni, An. maculatus, An. minimus*, *An. pampanai, An. rampae,* and *An. varuna*, were collected only in farm or forest sites (Tables 3–5). In village sites, LTs collected more mosquitoes (63.8%, 292/458) in IKD than in IDR (36.2%, 166/458) (p < 0.05, Monte Carlo Method) (Table 5). The lack of homogeneous LTs distribution, due to animal movements, and the bad weather impaired the collection of mosquitoes using LT in farm/forest sites, therefore no comparison was made between farm and forest sites. Additional file 1 reported the number of *Anopheles* mosquitoes collected outdoor and indoor using HDN and LT.

**Table 5:** Number and percentage of *Anopheles* collected using LT in village and farm and forest sites in IDR and IKD communes

Species	Ia DReh		Ia KDam		Total	%
	V	F	V	F		
*An. vagus*	69	–	156	11	236	47.9
*An. peditaeniatus*	38	–	67	–	105	21.3
*An. sinensis*	21	–	56	–	77	15.6
*An. tessellatus*	12	–	4	–	16	3.2
*An. monstrosus*	7	–	7	–	14	2.8
*An. aconitus*	7	–	–	6	13	2.6
*An. minimus*	–	–	–	7	7	1.4
*An. sawadwongporni*	–	–	–	7	7	1.4
*An. kochi*	5	–	–	–	5	1
*An. splendidus*	4	–	–	–	4	0.8
*An. barbirostris* s.l	1	–	–	2	3	0.6
*An. philippinensis*	2	–	–	–	2	0.4
*An. jamesii*	–	–	2	–	2	0.4
*An. dirus*	–	–	–	1	1	0.2
*An. varuna*	–	–	–	1	1	0.2
Total	166	–	292	35	493	100

*LT* light trap, *V* village sites, *F* farm and forest sites

### Seasonality

#### Mosquitoes collected in village sites using HDN, CS, and LT

In village sites, in both IDR and IKD communes, species differed depending on the season, except *An. sinensis* (49 and 51% collected in dry and rainy seasons, respectively, in IKD) (Table 6). *Anopheles aconitus*, *An. kochi*, *An. monstrosus*, and *An. sawadwongporni* were collected only in the dry season in IDR, and *An. monstrosus* in IKD; while *An. jamesii* and *An. philippinensis* were collected only in the rainy season in IKD (Table 6). In the dry season *An. barbirostris s.l.* (94%, 17/18), *An. jamesii* (89%, 8/9), *An. peditaeniatus* (79%, 292/369), *An. sinensis* (67%, 361/514), *An. splendidus* (92%, 12/13), and *An. tessellatus* (98%, 137/140) in IDR, and *An. peditaeniatus* (74% 265/358), and *An. tessellatus* (97%, 65/67) in IKD were more abundant compared to the rainy season; whereas *An. philippinensis* (67%, 4/6), and *An. vagus* (80%, 6,168/7,758), were more common in the rainy season in IDR, and *An. vagus* (81%, 5,662/6,958) in IKD (Table 6). Total *Anopheles* species collected in village sites were more common in the rainy season due to the high density of *An. vagus* (Table 6). When *An. vagus* was excluded, and comparisons were made between the other *Anopheles* mosquitoes, they are more abundant in the dry season at the two communes (82.5% in IDR, 1,130/1,370, and 69.2% in IKD, 662/957) (p < 0.05, Monte Carlo Method) (Table 6).

**Table 6:** *Anopheles* specimens collected during the dry and rainy season using CS, HDN, and LT, in village sites in Ia DReh and Ia KDam communes

Species	Ia DReh commune		Ia KDam commune	
	DS	RS	DS	RS
*An. aconitus*	44	–	–	–
*An. barbirostris*	17	1	–	–
*An. jamesii*	8	1	–	2
*An. kochi*	73	–	–	–
*An. monstrosus*	181	–	139	–
*An. peditaeniatus*	292	77	265	93
*An. philippinensis*	2	4	–	1
*An. sawadwongporni*	3	–	–	–
*An. sinensis*	361	153	193	197
*An. splendidus*	12	1	–	–
*An. tessellatus*	137	3	65	2
*An. vagus*	1590	6168	1296	5662
Total	2720	6408	1958	5957

*CS* collection around cattle shed, *HDN* human-baited double net trap, *LT* light trap, *DS* dry season, *RS* rainy season

#### Mosquitoes collected in farm/forest sites using HDN

In farm and forest sites, *An. philippinensis* were collected only in the dry season in IDR, and *An. aconitus* in IKD; while *Anopheles barbirostris s.l*., *An. maculatus*, *An. peditaeniatus*, and *An. vagus* were collected only in the rainy season in IDR, and *An. minimus* in IKD (Table 7). *Anopheles aconitus* (77.8%, 7/9) and *An. dirus* (76.5%, 65/85) were more common in the dry season in IDR, and *An. dirus* (67%, 2/3) and *An. peditaeniatus* (80%, 8/10) in IKD; whereas *An. sawadwongporni* (78.9%, 15/19) was more common in the rainy season in IRD, and *An. sawadwongporni* (75%, 9/12), *An. sinensis* (75%, 6/8) and *An. vagus* (57.4%, 39/68) in IKD (Table 7). In IDR, *Anopheles* mosquitoes were more abundant in the dry season (67.6%, 100/148), while in IKD they were slightly more common in the rainy season (53.1%, 60/113) (p > 0.05, Fisher Exact Test) (Table 7). High density of *An. dirus* was captured using HDN in farm/forest sites in both dry and rainy season*,* following by *An. tessellatus* in the dry season and *An*. *sawadwongporni* in the rainy season. *Anopheles vagus* was found in both dry and rainy season at village, and farm and forest sites (Table 8).

**Table 7:** *Anopheles* specimens collected during the dry and rainy season using HDN in farm and forest sites in Ia DReh and Ia KDam communes

Species	Ia DReh		Ia KDam	
	DS	RS	DS	RS
*An. aconitus*	7	2	1	–
*An. barbirostris*	–	2	–	–
*An. dirus*	65	20	2	1
*An. maculatus*	–	1	–	–
*An. minimus*	–	–	–	1
*An. monstrosus*	1	–	–	–
*An. pampanai*	–	–	–	1
*An. peditaeniatus*	–	1	8	2
*An. philippinensis*	1	–	–	–
*An. sawadwongporni*	4	15	3	9
*An. sinensis*	–	–	2	6
*An. tessellatus*	22	–	8	1
*An. vagus*	–	7	29	39
Total	100	48	53	60

*HDN* human-baited double net trap, *DS* dry season, *RS* rainy season

**Table 8:** HDN rates (number of mosquito/collector/night) for *Anopheles* species collected in village and farm and forest sites during the dry and rainy seasons in Ia DReh and Ia KDam communes

Species	Village^a^		Farm/forest	
	DS	RS	DS	RS
*An. aconitus*	–	–	0.080	0.020
*An. barbirostris* s.l	–	–	–	0.020
*An. dirus*	–	–	0.670	0.210
*An. kochi*	0.003	–	–	–
*An. maculatus*	–	–	–	0.010
*An. minimus*	–	–	–	0.010
*An. monstrosus*	0.003	–	0.010	–
*An. peditaeniatus*	0.028	0.015	0.080	0.020
*An. philippinensis*	–	–	0.010	–
*An. sawadwongporni*	–	–	0.070	0.240
*An. sinensis*	0.022	0.062	0.020	0.050
*An. splendidus*	0.003	–	–	–
*An. tessellatus*	0.006	–	0.300	0.010
*An. vagus*	0.086	0.284	0.290	0.460

*HDN* human-baited double bed net trap, *DS* dry season, *RS* rainy season

^a^Only outdoor trap was considered in the analysis

#### Observation on host-seeking activity of malaria vectors in humans (HDN) and cattle (CS)

**Fig. 2:**
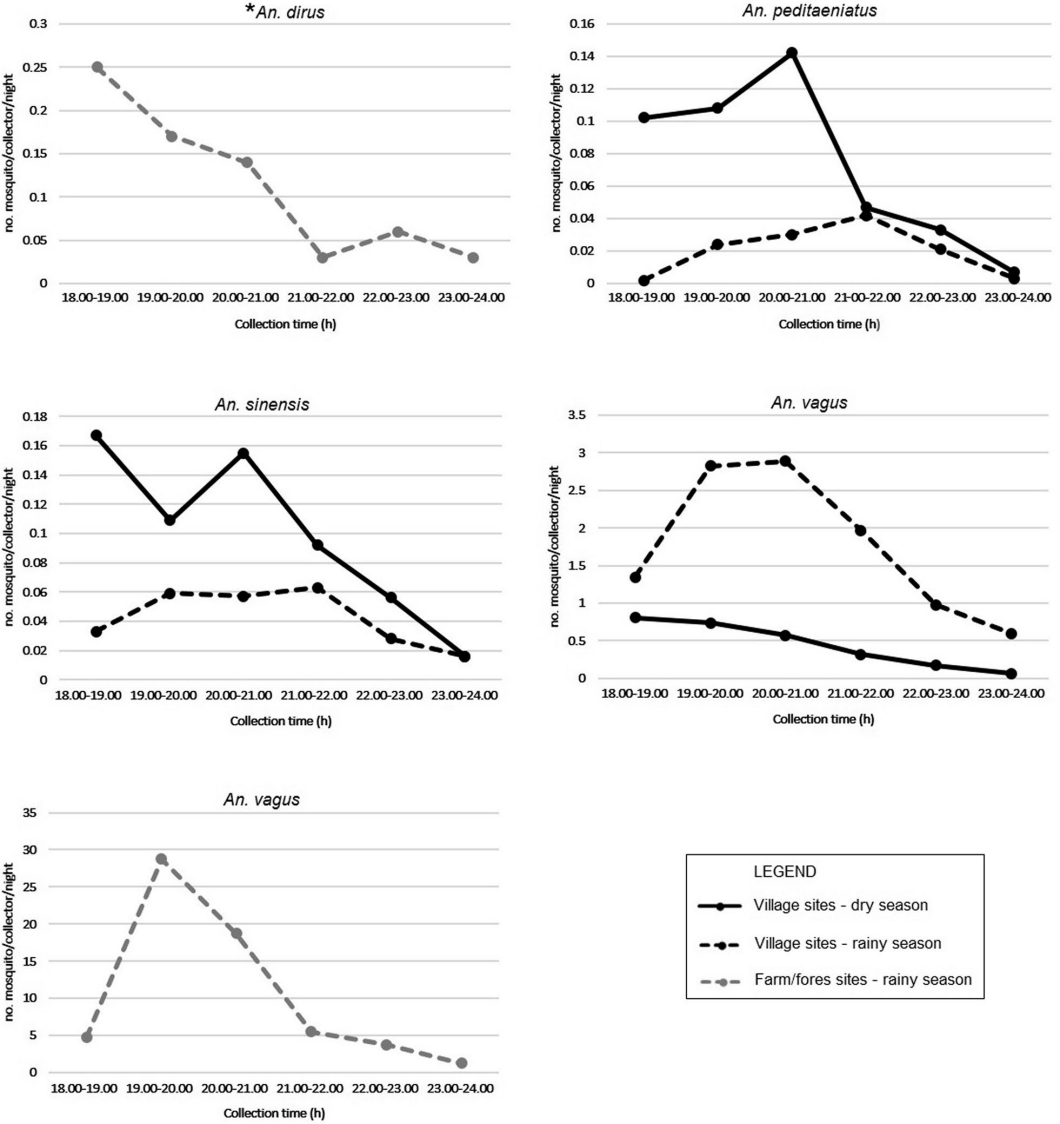
Peak of host-seeking activity of *Anopheles* species in Ia DReh commune, Krong Pa district, Gia Lai Province, Vietnam. *An. dirus was collected using HDN, the remaining samples were collected around CS. *HDN* human-baited double net trap, *CS* cattle shed

**Fig. 3:**
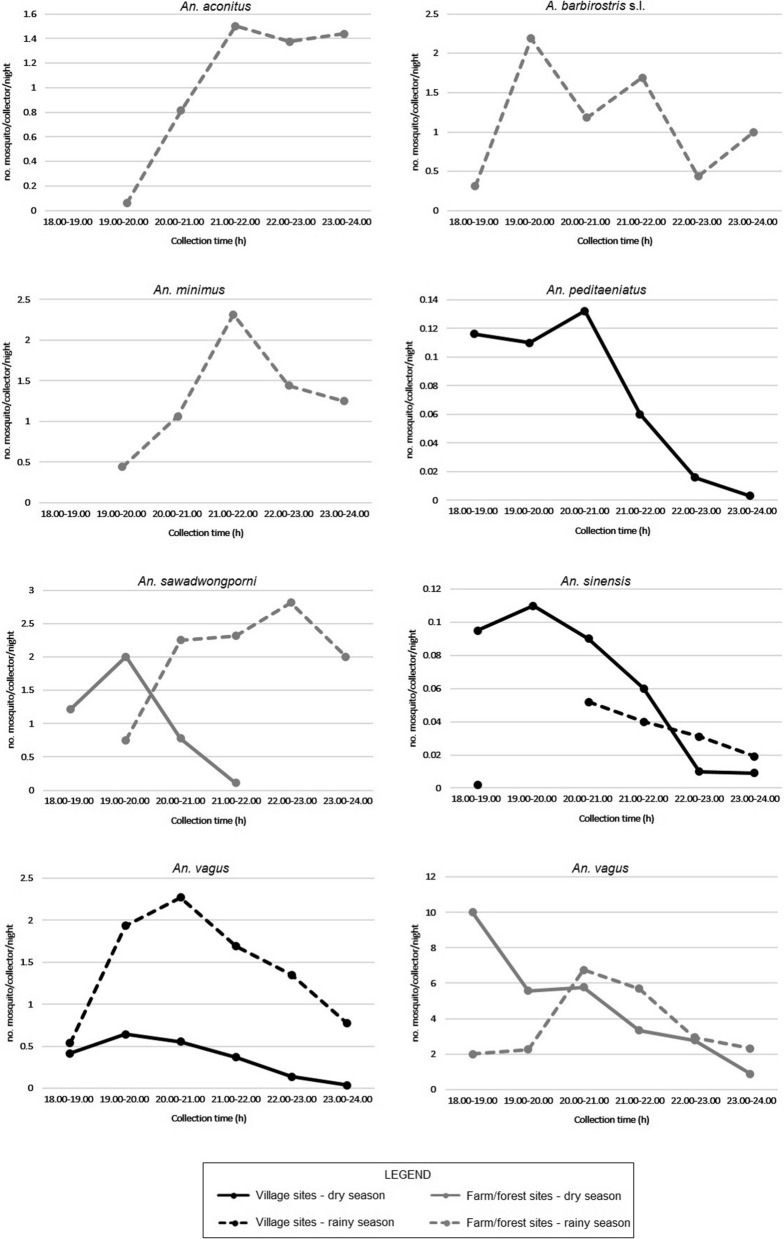
Peak of host-seeking activity of *Anopheles* species collected around CS in KDam commune, Ia Pa district, Gia Lai Province, Vietnam. *CS* cattle shed

The observation of host-seeking activity for primary and secondary malaria vectors in seeking humans and cattle are shown in the Additional files 2 and 3. Malaria vectors collected in high density are presented in Figs. 2 and 3.

In IDR commune, the peak of host-seeking activity for *An. dirus* collected using HDN in farm and forest sites occurred between 18.00 and 19.00 in the dry season (Fig. 2; Additional file 2). For mosquito species collected around CS in village sites, the peak occurred between 19.00 and 21.00 for *An. vagus*, between 19.00 and 22.00 for *An. sinensis*, and between 21.00 and 22.00 for *An. peditaeniatus* in the rainy season; in the dry season, the peak of host-seeking activity for *An. sinensis* and *An. vagus* occurred between 18.00 and 19.00, and between 20.00 and 21.00 for *An. peditaeniatus* (Fig. 2; Additional file 2). The peak of host-seeking for *An. vagus* collected in farm and forest sites during the rainy season was between 19.00 and 20.00 (Fig. 2; Additional file 2.

In village sites in IKD commune, the peak of host-seeking activity was between 20.00 and 21.00 for *An. sinensis* and *An. vagus* during the rainy season; and between 19.00 and 20.00 for *An. An. sinensis* and *An. vagus*, and between 20.00 and 21.00 for *An. peditaeniatus* in the dry season (Fig. 3; Additional file 3). In farm and forest sites of IKD, the peak of host-seeking activity was between 19.00 and 20.00 for *An. barbirostris* s.l., between 20.00 and 21.00 for *An*. *vagus*, between 21.00 and 22.00 for *An. aconitus* and *An. minimus*, and between 22.00 and 23.00 for *An. sawadwongporni* in the rainy season. In the dry season, the peak for *An. vagus* occurred between 18.00 and 19.00, and for *An. sawadwongporni* between 19.00 and 20.00 in the dry season (Fig. 3; Additional file 3).

## Discussion

### *Anopheles* diversity and seasonality

Herein, the two primary malaria vectors, *An. dirus* and *An. minimus* were found at the two study sites of very low malaria transmission in Gia Lai Province, along with 9 secondary malaria vectors: *An. aconitus, An. barbirostris* s.l.*, An. harrisoni, An. maculatus, An. peditaeniatus*, *An. philippinensis, An. sawadwongporni*, *An. sinensis,* and *An. vagus*. Overall, 19 *Anopheles* species (14 taxa in IDR, and 18 taxa in IKD) were identified at the two study sites. From the same province, in Kong Chro district (40 km from IDR and 70 km from IKD), Garros et al. [28] collected 11 *Anopheles* species, which included *An. dirus* s.l.*, An. maculatus* s.l., and *An. minimus* s.l.. Identification of these mosquito specimens were based on morphology only, and thus it was not possible to identify which members of each species complex were found in their study. All *Anopheles* species found from the study by Garros et al. [28] were collected from the IKD and IDR sites in this study, except *An. jeyporiensis*, and *An. nivipes*. By combining the *Anopheles* species identified by Garros et al. [28] and those collected herein, a total of 21 *Anopheles* species have been identified from Gia Lai Province.

*Anopheles* diversity was greater in the forest sites (12 taxa in village sites, and 18 taxa in forest sites). *Anopheles dirus* and *An. minimus,* and the secondary malaria vectors, *An. aconitus*, *An. barbirostris* s.l., and *An. sawadwongporni* were more common in farm/forest sites. Although previous studies have incriminated *An. aconitus* and *An. sawadwongporni*, and other *Anopheles* species (*An. harrisoni*, *An. maculatus*, *An. pampanai*, *An. peditaeniatus An. philippinensis, An. sinensis*) in maintaining malaria transmission in rural areas of the country [10, 29], still little is known about the role of secondary vectors in residual malaria transmission in rural areas.

*Anopheles dirus* and *An. minimus* were found only in forest sites. This might indicate the prevalence of ‘external’ malaria infection (infection outside the village) [8] observed in workers associated with forest sites in IDR (IDR health station, pers. comm.). Rainforests and secondary forests or plantations (rubber, fruits) provide appropriate breeding sites for *An. dirus* [30, 31]. The farm and forest sites in both communes are around 3 km and 10 km (IKD and IDR, respectively) away from the villages, and outside the flight range (approximately 1.5 km) for *An. dirus* [30]*.* Therefore, the long distance between the breeding sites and the villages may be the reason for the absence of *An. dirus* in village sites. The breeding sites for *An. minimus* are usually ubiquitous [32], but perhaps in IKD this species is a habitat specialist, and thus found only in the farm/forest sites. Also, these sites can offer more host variety (i.e., cattle, chicken, dog). However, ecological studies are needed to clarify the ecology of *An. minimus* in Gia Lai Province.

*Anopheles maculatus* and *An. sawadwongporni* were the only vectors molecularly identified in the Maculatus Group. *Anopheles sawadwongporni* was the most collected species of the Maculatus Group in this study, making up 1.3% (240/18,835) of all collected species, and only a small number of *An. maculatus* (IDR, n = 1; IKD, n = 5) were found in and around farm/forest areas. These two mosquito species are considered as secondary malaria vectors throughout Vietnam [10]. *Anopheles maculatus* was the dominant species collected in the southern province of Dak Nong [33] (170 km from IDR, 200 km from IKD), and was co-implicated with *An. sawadwongporni* as the primary vectors in Hang Chuon village, southwestern Quang Binh Province [10] (495 km from IDR, 465 km from IKD). The vector status of *An. maculatus* and *An. sawadwongporni* in IDR and IKD is still unclear.

*Anopheles vagus* was the dominant species*,* followed by *An. sinensis* and *An. peditaeniatus* of both communes*.* Due to its ability to survive in small pools of turbid and organically polluted water [34], *An. vagus* has been found in high densities in countries of Southeast Asia (SEA) [8, 10, 28], including *An. vagus* positive for circumsporozoite protein (CSP) in mosquitoes in Bangladesh [35, 36]. However, there is no published information about the vector status of this species in Vietnam. *Anopheles peditaeniatus* have been found to be positive for CSP in Indonesia [37] and Thailand [38], and *An. sinensis* was involved with malaria transmission in China [39] and epidemics in Korea [40]. *Anopheles peditaeniatus* and *An. sinensis* were found to be positive for CPS in Quang Binh Province, Vietnam [10], with *An. sinensis* considered the major malaria vector for the province. The adaptable *An. vagus* has been observed in different environments [10, 34] and because of its opportunistic and zoophilic behaviour [8, 10] limited research has been carried out on this species. *Anopheles vagus* appears not to play an important role in malaria transmission at the two study sites.

A high abundance of mosquitoes is expected in the rainy season, where more breeding sites are available and conditions are favourable for mosquito development. Indeed, more mosquitoes were collected during the rainy season compared to the dry season in this study, similar to reports from Thailand and Bangladesh [30, 41]. Similarly, an increased abundance of *An. dirus* and *An. minimus* has been observed in Vietnam during the rainy season between September and November [42]. Herein, *An. minimus* was present only during the rainy season, however, when combining the collections from the village and farm/forest areas of the two sites (IDR and IKD) most of the *An. dirus* (74.4%, 67/90) were collected in the dry season. Rain was especially intense in the forest sites in both communes during the rainy season, and apparently flooded the breeding sites of *An. dirus* and other species that have more suitable habitats in the forest in IDR. On the other hand, *An. minimus* and the dominant *An. vagus* were more common during the rainy season, probably due to the temporary breeding sites being more available in IKD for their development. Nevertheless, *An. dirus* was still found in the rainy season, as well as secondary malaria vectors collected throughout both seasons. This suggests that favourable conditions for vector development exist year-round, which ensures the maintenance of malaria parasites. Consequently, the epidemiologic triad of host (human, animal), agent (*Plasmodium* + *Anopheles*), and environment conditions contribute to the maintenance of malaria transmission in the study areas.

### Collection and behaviour of malaria vectors

Due to logistical limitations, the distribution of traps in the two communes and the number of collections were not homogeneous. The limitations included difficulty in setting up traps, arranging and maintaining cows in farm and forest sites, and loss of LTs. Unfortunately, many traps were knocked over and found empty, probably because of animal movement during the night. Also, during the collection, the weather impacted sample collection, with heavy rain and flooding which made it difficult to collect the mosquitoes at the communes. Nonetheless, specific comparisons of the mosquitoes collected for each collection method was carried out.

Studies of malaria vectors in SEA have used a variety of methods to collect mosquitoes, including human baits (human landing catches—HLC and HDN) [8, 10, 15, 24, 43], animal baits (buffalo, cattle) [8, 10, 15, 33], and LTs [33, 44–46] to collect mosquitoes. Light traps are a cost-effective method for mosquito collection; however, the efficacy varies by location [45, 47]. Another cost-effective method for the collection of vector species is HDN, an alternative to HLC, which eliminates exposing collectors to mosquito bites [24]. Herein, a high density and diversity of mosquitoes were collected using CS. In other studies, similar results were reported using CS, with a high diversity of mosquitoes collected including primary and secondary malaria vectors [10, 28, 48]. Despite the simultaneous collection using human (HDN) and cattle bait (CS), it was not possible to calculate the anthropophilic index due to the heterogeneous distribution of traps and number of collections, and the prominent zoophilic behaviour observed of all *Anopheles* species, except *An. dirus.*

*Anopheles dirus* was observed to be the most anthropophilic species in this study and is consistent with other studies conducted in SEA [13, 30, 31, 33, 49]. However, there are reports of *An. dirus* with strong zoophilic behaviour in Kanchanaburi Province, Thailand [50] suggesting some variance in behaviour across SEA. *Anopheles minimus,* together with many secondary vectors identified in this study, showed zoophilic behaviour, which has been similarly observed in other regions in Vietnam and neighbouring countries [10, 15, 51–54]. *Anopheles minimus* host-seeking behaviour is similarly variable with anthropophilic behaviour observed in Khanh Hoa Province (Vietnam) and Vientiane Province (Laos) while Van Bortel et al. [32] reported heterogeneity in host choice. There are reports *An. minimus* are attracted to cattle when abundant, but are attracted to humans when cattle are scarce [15, 32, 55]. In this study *An. minimus* was found exclusively in farm/forest areas in IKD, where there were many types of host, such as cattle, chicken and dogs. The close proximity of cattle to household areas has been associated with an increase in malaria prevalence [56, 57] suggesting a relationship to host-seeking behaviour and horticulture practices. A future study of blood meals in *An. minimus* is required to better understand the impact of host choice on vector survival and reproduction in areas of malaria transmission.

Do Manh et al. [10] conducted a malaria survey in Quang Binh Province (Vietnam) using paired collections of buffalo and humans (with 15 m of distance between them), and observed that 97.4% (4,489/4,610) of the anopheline collected were attracted to buffalo and only 2.6% (121/4,610) to humans using HLC. The authors suggested that animals (buffalo/cattle) around human settlements may play a zooprophylactic role [10]. In this study, it was observed that anopheline were more attracted to animals than human, similar to Do Manh et al. findings [10]. To reduce vector density and longevity, Do Manh et al. [10] proposed a strategy of maintaining animal baits (cattle, buffalo) around human habitation and implementing barrier spraying to supplement other intervention methods such as IRS and the use of LLIN.

The early host-seeking time of 18.00 to 19.00 observed for *An. dirus*, *An. sinensis* and *An. vagus* coincided with human activities outdoors. This study implies that the use of LLIN after 21.00 will have limited impact on reducing *Anopheles* bites. Villagers and forest workers usually go to bed after 21.00 (TQN pers. obsv.), therefore, the use of LLIN in areas where vectors bite outdoors and/or early in the evening when people are still active will not be very effective [58]. Additionally, some bed nets appeared worn with large holes or gashes, limiting their ability to protect users. LLINs are a critical component of the NMCP, however, there are reports of limited use despite near-universal availability [11, 12, 22]. Perhaps, an alternative to support the strategies to prevent malaria transmission could include the distribution of repellent or repellent clothing (treated coveralls) to the villagers-workers in addition to the LLINs as cost-effective methods for protection against malaria vectors. Moreover, additional research based on practical personal protection tools (i.e., those do not require behaviour change and are easy to use) is required to eliminate residual malaria transmission [5] in these areas.

## Conclusions

Nineteen *Anopheles* species were identified at the two study sites in Gia Lai Province, including two primary malaria vectors, *An. dirus* and *An. minimus* and 9 secondary malaria vectors. During the course of this study, *An. dirus* displayed anthropophilic preferences, whereas *An. minimus* and a number of the secondary vectors displayed zoophilic preferences for host. The observed results provided information on the diversity, seasonal prevalence and behaviour of *Anopheles* in the study sites. Moreover, the peak (18.00 to 23.00) *Anopheles* host-seeking activity was observed in this study highlighting the future need to focus on *Anopheles* behaviour in this region of the central highlands of Vietnam.

## Supplementary Information

**Additional file 1.** Number of *Anopheles* specimens collected indoor and outdoor using HDN and LT in village and farm and forest sites in Ia DReh commune, Krong Pa district, and in Ia KDam commune, Ia Pa district, Gia Lai Province, Vietnam.

**Additional file 2.** **A.** HDN rates (number of mosquito/collector/night) for malaria vectors collected in village and farm/forest sites during dry and rainy seasons in Ia DReh commune, Krong Pa district, Gia Lai Province, Vietnam. **B.** CS rates (number of mosquito/collector/night) for malaria vectors collected in village and farm/forest sites during dry and rainy seasons in Ia DReh commune, Krong Pa district, Gia Lai Province, Vietnam

**Additional file 3.** **A.** HDN rates (number of mosquito/collector/night) for malaria vectors collected in village and farm/forest sites during dry and rainy seasons in Ia KDam commune, Ia Pa district, Gia Lai Province, Vietnam **B.** CS rates (number of mosquito/collector/night) for malaria vectors collected in village and farm/forest sites during dry and rainy seasons in Ia KDam commune, Ia Pa district, Gia Lai Province, Vietnam.

## Data Availability

All data generated or analyzed during the current study are included in this published article.

## Abbreviations

CDC: Center for Disease Control and Prevention
CSP: Circumsporozoite protein
CS: Collection around cattle shed
HDN: Human-baited double net trap
HLC: Human landing catches
IDR: Ia DReh commune
IKD: Ia KDam commune
ITN: Insecticide-threated net
LLIN: Long-lasting insecticidal net
LT: Light trap
NMCP: National Malaria Control Programme
SEA: Southeast Asia
UV: Ultraviolet
